# Nanoengineering in Cardiac Regeneration: Looking Back and Going Forward

**DOI:** 10.3390/nano10081587

**Published:** 2020-08-12

**Authors:** Caterina Cristallini, Emanuela Vitale, Claudia Giachino, Raffaella Rastaldo

**Affiliations:** 1 Institute for Chemical and Physical Processes, IPCF ss Pisa, Italian National Research Council (CNR), 56126 Pisa, Italy; caterina.cristallini@cnr.it; 2 Department of Clinical and Biological Sciences, University of Torino, 10043 Orbassano, Torino, Italy; emanuela.vitale@unito.it (E.V.); claudia.giachino@unito.it (C.G.)

**Keywords:** nanomaterial, extracellular matrix, cardiac cells, cardiac regeneration, hybrid materials, tissue engineering

## Abstract

To deliver on the promise of cardiac regeneration, an integration process between an emerging field, nanomedicine, and a more consolidated one, tissue engineering, has begun. Our work aims at summarizing some of the most relevant prevailing cases of nanotechnological approaches applied to tissue engineering with a specific interest in cardiac regenerative medicine, as well as delineating some of the most compelling forthcoming orientations. Specifically, this review starts with a brief statement on the relevant clinical need, and then debates how nanotechnology can be combined with tissue engineering in the scope of mimicking a complex tissue like the myocardium and its natural extracellular matrix (ECM). The interaction of relevant stem, precursor, and differentiated cardiac cells with nanoengineered scaffolds is thoroughly presented. Another correspondingly relevant area of experimental study enclosing both nanotechnology and cardiac regeneration, e.g., nanoparticle applications in cardiac tissue engineering, is also discussed.

## 1. Introduction: Clinical Need

Heart disease is a priority area for regenerative medicine, accounting for more than 30% of all deaths including the main three clinical syndromes, ischaemic heart disease (IHD), stroke, and peripheral ischaemic disease. Even though frequency in the developed countries tends to decrease, this is balanced by its rise in the developing world. Indeed, the World Health Organization (WHO) reported that, in 2016, ischaemic heart disease was still the most common cause of mortality worldwide accounting for about 9.4 million deaths [1]. In a large portion of patients, IHD first shows itself as an acute myocardial infarction (AMI), conceivably resulting in sudden death or causing heart failure (HF) during the subsequent chronic stage. Indeed, infarct of the myocardium represents the biggest reason of HF all over the world, and HF is heavily related to the quantity of compromised tissue portion subsequent to acute ischaemia/reperfusion [2]. The scarce regenerative potential of the human myocardium causes a failure in reestablishing the correct cardiomyocyte (CM) cell numbers and in counteracting the formation of scarred tissue, a situation often leading to arrhythmic events, altered pumping activity and HF. In spite of current pharmacological approaches and interventional therapies, the morbidity and mortality of patients with seriously compromised ventricular function following AMI remain very high. Ventricular assist devices represent a great accomplishment of current medicine, thanks to their ability to supply temporary functional aid to failing organs on the road to transplantation. They do not represent, however, a permanent answer to the problem, as they show supplementary disadvantages connected for example with augmented haemostatic occurrence, bleeding events and risks of infection. Another critical aspect is that these mechanical devices are not endowed with the ability to adapt to the changeable patient’s metabolic requests. At present, the unique long-lasting therapeutic intervention for severe HF remains organ transplantation, however the actual donation rates fall widely below population needs, including nations where transplant rates are the highest.

As an answer to this unmet need, in the last twenty years possible approaches pointing to regenerate the damaged heart consequent to AMI have represented object of focused fundamental [3] and translational research [4]. Regrettably, therapeutic options in the area of cardiac regeneration, widely founded on cell implantation, have thus far given up disappointing results [5,6].

To overcome the limit of the therapeutic effectiveness of stem cells (SCs) transplantation (i.e., low survival, retention/engraftment, homing, and differentiation) some strategies have been attempted such as preconditioning, genetic modification, co-transplantation with bioactive factors, and tissue engineering aimed at modifying cell behaviour and favouring cell differentiation, as already reviewed by Chen’s group in the case of mesenchymal stem cells (MSCs) [7]. Tissue engineering is defined as “the application of principles and methods of engineering and life sciences toward the fundamental understanding of structure–function relationships in normal and pathological mammalian tissues and the development of biological substitutes to restore, maintain or improve tissue function” [8]. In particular, “myocardial tissue engineering” (MTE) is considered one of the most promising strategies to efficiently treat cardiac disorders and involves various approaches for the generation of three-dimensional myocardium-like constructs that should provide mechanical support to damaged myocardial tissue and mainly instructs stem cell, both seeded and in situ recruited, towards a correct morphogenesis.

However, these strategies should be still optimized to achieve clinical safety. On the other hand, acellularized or cellularized nanomaterial-based therapy provides promising opportunities to overcome the limitations related to the SCs implantation and tissue regeneration.

Various studies demonstrated that scaffolds provided not only a suitable mechanical support to cell culture, but they can also regulate SC behaviour by favouring SC survival, proliferation and differentiation, and providing a guide for tridimensional (3D) tissue reconstruction [9,10,11]. In fact, various studies showed that differentiation occurred either in the presence [12,13,14,15,16] or in the absence [9,17,18,19] of external stimuli when SCs have been cultured on biomaterials. In particular, the exclusion of exogenous factors allowed to demonstrate that an enhanced differentiation could be driven by the physical, chemical, and biological properties of biomaterials.

A strategy capable of stimulating the regeneration of myocardium accompanied by a protective effect to prevent its maladaptive remodeling would lead to an efficient therapeutic action. To deliver on these promises for heart disease, one must have reliable means for working at the nanoscale, exploiting nanoengineering to generate complex tissues like the myocardium.

Here, we overview the recent nanotechnological applications combined with tissue engineering to the scope of mimicking a complex tissue like the myocardium and its natural extracellular matrix (ECM) and highlights how the versatility of nanomaterials can be exploited to favour the interaction of relevant stem, precursor, and differentiated cardiac cells with nanoengineered scaffolds to advance cardiac regeneration.

## 2. Cardiac ECM as a Guide to Nanoengineering

**Figure 1:**
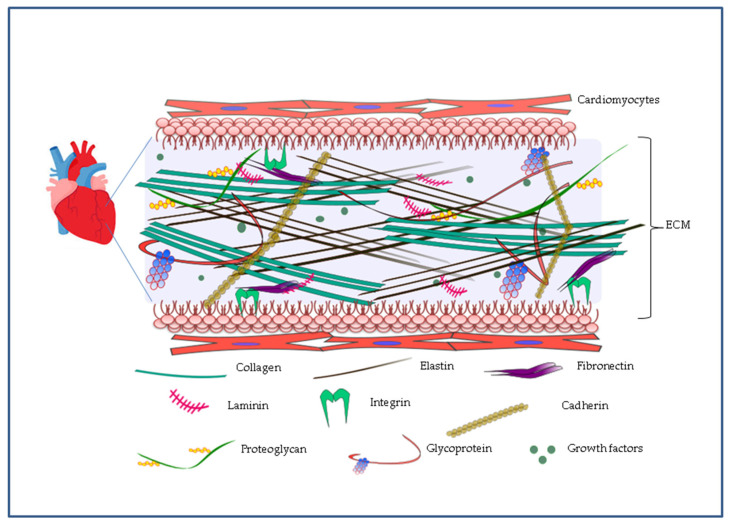
Representation of the complex tridimensional structure of the cardiac extracellular matrix (ECM). ECM is made of a dense network of proteins such as elastin, collagens, proteoglycans and glycoproteins. Through proteins such as fibronectins, laminins, cadherins and integrins, ECM plays a key role in cellular functions such as cell adhesion and growth thanks to the presence of soluble substances including growth factors.

In a developing organ, the 3D network constituted by ECM drives its initial complexity. Thus, reproducing the ECM will help to recapitulate complexity in tissue engineering. ECM is endowed with a complicated arrangement of many structural proteins with nanoscale features including collagen and fibronectin, proteoglycans, and glycoproteins as well as soluble substances including growth factors (Figure 1). It not only provides a framework for cell to ECM and cell to cell interactions, but also regulates some important cellular functions such as adhesion and growth. During the past several years, an improved ability to manufacture a genuine biomimetic microenvironment came from production of innovative synthetic nanosystems and from their assembly into current macro- and microscale matrices. More recently, it became apparent that nanocomposites can help in overcoming matrix limitations in current biomaterials [20].

The elastic heart wall consists of compacted fibrillar collagen and elastin bundles forming a tight network which ranges from ten to several hundreds of nanometers across. Fibronectin and laminin, proteins with nanoscale features and adhesive properties, completely cover this elastic mesh, allowing cells to surface adhere through integrins and cadherins (Figure 1) [21]. Conceptually, the myocardial ECM can be classified into elements residing in the interstitial matrix (collagen I, collagen III, and fibronectin) and residing in the highly organized sheet-like layer of ECM known as basement membrane (collagen IV, V, VII, X, and XIV and laminin) which plays a central contribution in sarcomere assembly through interactions with integrins [22].

A pioneering study by Ott and colleagues in 2008 emphasized the importance of the ECM in engineering a bioartificial rat heart by guiding morphological organization and essential physiological functions [23]. This study started with the detergent-based decellularization of adult rat hearts through coronary perfusion, which allowed for the preservation of the underlying ECM. Upon reseeding with cells of the myocardial and endothelial lineages, the ECM sustained self-organization leading to restored pump function. On the other hand, when the same cells were seeded on a flat scaffold, a random cellular distribution was observed and CMs lost their elongated morphology, resulting in a compromised physiological function.

Another group demonstrated that also SCs differentiate towards myocardial phenotype when cultured on decellularized human cardiac ECM slices, obtained from cardiac samples taken from patients. There was an increased expression of cardiac transcription factors and sarcomeric proteins in both embryonic stem cells (ESC) and induced pluripotent stem cells (iPSCs), while ECM failed to induce MSC differentiation [24]. On the contrary, Matrigel and Geltrex did not promote the expression of cardiac markers in any type of cells [24].

Stem cell niches provide spatial and temporal signals, which control SC behaviour such as self-renewal, differentiation and therefore cell fate and indeed, niches are composed of ECM components, soluble factors, and supportive cells [25].

These works demonstrated that microenvironment is essential to induce an efficient differentiation of SCs towards cardiac lineage, thereby engineered nanostructured scaffolds must recreate the characteristics of the native tissue by mimicking the physical and biological features of the ECM to achieve the regeneration of cardiac tissue.

## 3. Nanopatterning Technologies for Cardiac Tissue Regeneration

### 3.1. Micro-Nanotopography

**Figure 2:**
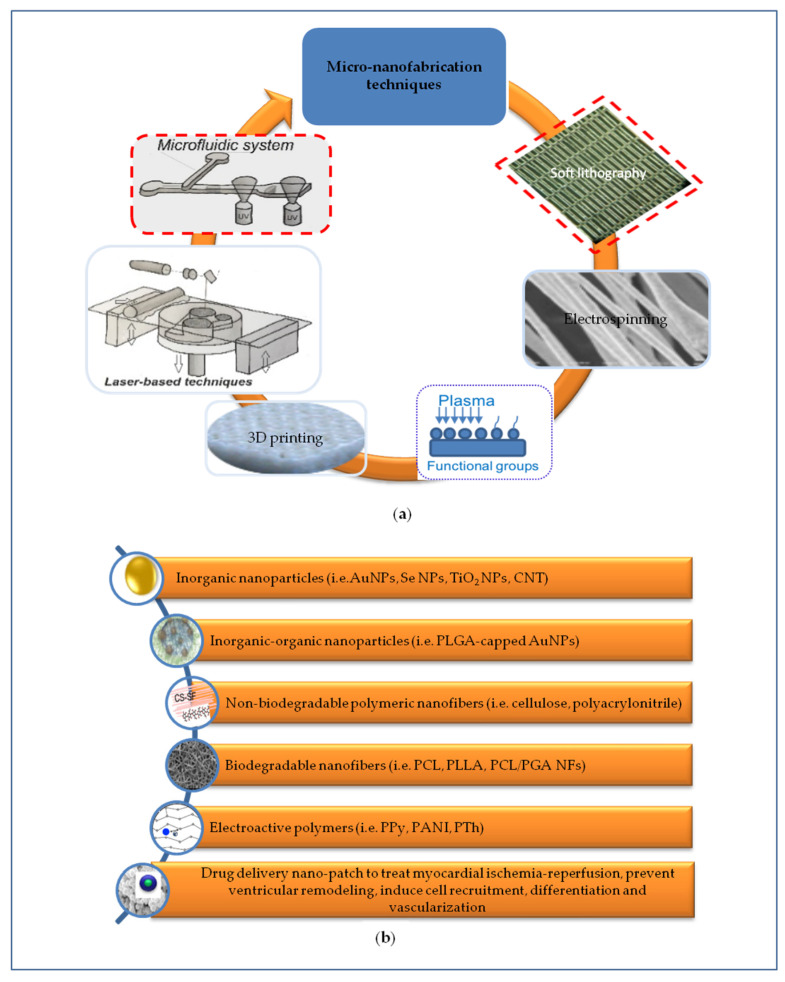
Representation of different micro-nanoengineering approaches to develop scaffolds recapitulating the complex signals necessary for cardiac regeneration. (**a**) Micro-nanofabrication techniques are crucial to produce scaffolds mimicking the anisotropic micro-nanostructure and mechanical properties of native cardiac ECM; (**b**) Micro-nanostructured scaffolds based on non-biodegradable and/or biodegradable polymers were further implemented by incorporating electrically conductive nanomaterials to provide relevant electro-mechanical stimulation. Implementing cardiac patch with drug delivery systems using different methods can help to treat simultaneously myocardial ischemia-reperfusion, to prevent left ventricular remodeling and to induce cardiac regeneration processes.

The optimization of surface micro and nano topography mimicking anisotropic structure of the myocardium is fundamental to provide a spatial interface to control adhesion, elongation, ordered disposition and myocardial commitment of SCs. Some representative examples of nanomaterials and their relevant biological effects are summarized in Table 1. Micro and nanofabrication methods allowed to transfer the information at these scales in a controlled, predefined, and versatile way (Figure 2a) [26]. Optical lithography was used to direct the attachment of cardiac cells cultured on surfaces, having a patterning with 20-μm wide lines, to control the morphology and microtubule density of cardiac myocytes [27]. Methacrylated tropoelastin (MeTro) coating by means microfluidic method allowed the neonatal rat CMs attachment and beating [28]. Soft lithography consented to fabricate arrays at level of nanoscale using poly(ethylene glycol) (PEG) hydrogel [29]. Nanofabric mimicking ECM and containing fibronectin, laminin, collagen type I and collagen type II was obtained by soft lithography. Through this technique a nanotopography with nanopillars and nanogrooves allowed the control of SC differentiation including beating CMs. This is a promising but still very immature study which faces many problems that need to be addressed (for example homogeneity) [30].

Laser-based techniques are alternative and promising approaches to modify a variety of materials by writing directly the predefined surface geometry at micro and nano scale. Laser ablation was used on biological scaffold (agarose) inducing improved attachment and differentiation of C2C12 myoblasts [31]. The same method was used for the production of multi-layered elastomeric scaffolds having an accordion-like honeycomb structure in order to replicate the native myocardium microenvironment and anisotropy of the left ventricular tissue [32,33].

Plasma surface functionalization was used in combination with other techniques to improve the hydrophilicity of a surface and spreading, myofibril development and gene expression of primary neonatal rat CMs and human iPSC-CMs on synthetic, such as poly(lactic-co-glycolic acid) (PLGA), poly(ε-caprolactone) (PCL), poly(3-hydroxybutyrate) (PHB), and nanofibers (NFs), or biological substrates, such as fibronectin, for cardiac tissue engineering [34,35,59].

Recently, a great interest was focused on the use of electrospinning to develop scaffolds based on electrospun NFs with controlled diameters able to replicate the myocardium ECM. Electrospinning offers the potential of fabricating composite materials of natural and synthetic polymers that can be implemented with gold nanoparticles (NPs) [36,37] and carbon nanotubes (CNT) [38] to improve the conductivity of the construct as well with peptides [39] and growth factors [40] to improve the remodeling and viability of CMs. This technique was even applied with living cells, and a recent study reported primary neonatal CMs electrospinning to realize living constructs while preserving their functionalities [39].

Additive manufacturing, known as 3D printing, has recently rapidly evolved as a remarkable approach in tissue engineering to realize scaffolds, devices and 3D structure including complex organ like heart [18]. Bioprinted constructs were prepared using alginate and human CM progenitor cells, demonstrating cell viability, retention of cardiac lineage, enhanced expression of genes and early cardiac transcription factor sarcomeric protein. A further example of bioprinted scaffold was obtained by combining hyaluronic acid/gelatin with human fetal CM progenitor cells, with the aim to serve as a cardiogenic patch. A cardiac patch produced using cell printing of human umbilical vein endothelial cell and human MSCs on a defined surface, after in vivo implantation in a murine model, showed increased vessel formation and integration into the murine vascular system, with a possible benefit in AMI treatment. Bioprinted cardiac constructs using alginate and human coronary artery endothelial cells were used for in vivo studies offering a high cell viability and distribution, and a good agreement with the in vitro results in terms of impedance or mechanical behaviour [22]. PCL printed scaffolds using a microextrusion 3D printer were produced by introducing wirelessly controlled electrical stimulators, the effect of electrical stimulation improved the attachment and induced differentiated actin cytoskeletal structures. PCL 3D printed scaffold modified with CNT was used to improve H9C2 viability respect to not modified scaffold [41]. Stereolithography was used for fabricating an innovative scaffold, based on photoactive gelatin polymer, where CMs, smooth muscle cells and endothelial cells (ECs) derived from human iPSCs were seeded [42]. This human iPSCs–derived cardiac muscle patch was evaluated in a murine model of MI showing improved cardiac function and reduced infarct size after AMI [43]. Moreover, a 3D poly(ethylene glycol) diacrylate (PEGDA) based scaffold obtained by micro-stereolithography promoted human cardiac progenitor cell differentiation and orientation, 3D spatial ordered orientation, and activation of the expression of α-sarcomeric actinin (α-SA) and connexin 43 (Cx43).

In combination with micro-nanofabrication, a relevant advancement is represented by the microfluidic technology that has the potential to overcome the limitations of static culture systems by reproducing complex cardiac human disease models in vitro, developing drug screening platforms and controlling physico-chemical and electro-mechanical conditions of specific cell co-cultures (according the approach named “heart on a chip”) [60,61].

### 3.2. Nanomaterials for Cardiac Patches

The use of NPs to modify the scaffold surface is an emerging strategy to improve and implement various functionalities for complex engineered tissues.

Nanoparticles mimic the physiological nanometric size of ECM components of tissues and their reduced size accompanied by large surface-to-volume ratio share similarity with peptides or small proteins. Mimicking the architectural organization of the native ECM, nanomaterial acquires also some functional properties of ECM. Nanostructures can be incorporated into organic or inorganic materials, thus improving the properties of the resulting composite nanomaterials and acquiring instructive cues (Figure 2b).

#### 3.2.1. Inorganic Nanoparticles

The incorporation of inorganic nanoparticles, having suitable properties of biocompatibility and electro-conductivity, into a scaffold can provide a series of advantages to a cardiac patch including: (a) increased electro-conductive properties, (b) improved cell compatibility, (c) a biomimetic surface topography, and (d) increased mechanical stability.

AuNPs represents an emerging class of nanosystems for cardiac tissue engineering due to its numerous advantages in terms of anti-cardiac hypertrophy effect, biocompatibility, electrical conductivity, and chemical stability. AuNPs present high electrical conductivity, variety of geometry (i.e., nanospheres, nanorods, nanowires), intrinsic optical properties, ease of surface functionalization and a suitable cytocompatibility. For this reason, AuNPs were incorporated into different biological and synthetic matrices and their physico-chemical and biological effects on scaffolds after seeding with cardiomyoblasts or CMs were evaluated, as reported in the following studies.

Nanomodification with inorganic particles (gold particles) of ECM/silk fibroin (SF) and MSC offered a new patch suitable for cardiac tissue engineering. The distribution of homogeneous AuNPs demonstrated to provide favourable conductivity, in vitro enhanced cell compatibility, retention of CM survival and in vivo decreasing of infarct size from 89% to 65% in the control group [44].

AuNPs were conjugated to cholecyst derived xenogenic ECM, by means 1-ethyl-3-(3-dimethyl aminopropyl)-carbodiimide/N-hydroxysuccinimide method. The modified scaffold allowed the growth and proliferation of the cardiomyoblasts (H9c2 cells) and the introduction of AuNPs provided a conductivity property to the scaffold useful for improving cell communication and controlling the polarity of cells [45].

The incorporation of gold nanowires (NWs) within alginate scaffolds increase the electrical signal propagation among pore walls of alginate and improve electroconductivity and functional of cardiac cells towards a suitable organization of cardiac tissue [46].

A combination of AuNPs and laponite NPs loaded into myocardial ECM improved cell compatibility and phenotypes maturation of cardiac specific proteins, enhancing the cell expression of cardiac specific markers, such as cardiac troponin (cTn) I and Cx43 [47].

Recently, an interesting class of nanoparticles, Selenium NPs, was investigated for its ability to transfer electrical signals in seeded scaffolds. Selenium NPs are naturally present in human body with benefit in terms of biocompatibility. Furthermore, many cardiac patients have low level of selenium in blood, thus their use can be beneficial also for their therapeutic potential after cardiac surgery.

Selenium NPs, obtained by a novel one-pot green synthesis, were used to modify chitosan film; a recent study confirmed the potential of substrate modified with these nanoparticles to provide electrical conductivity (0.0055 S cm^−1^) in order to promote rapid electrical coupling between CMs, and enhance mechanical properties in agreement with those of native myocardium [48].

Among different categories of nanoparticles, titanium dioxide (TiO_2_) NPs have received great attention in biomedical field for their biocompatibility, good physico-chemical properties, biological stability, and protein adsorption. The introduction of TiO_2_NPs into a hydrogel matrix can regulate the mechanical, swelling and degradation behavior by improving the properties of hydrogels for use in tissue engineering.

In a recent study, TiO_2_NPs were homogenously dispersed in PEGylated chitosan hydrogel demonstrating their capability of improving mechanical and swelling properties and also inducing cell adhesion and organization of CMs suggesting an efficient use of these nano-modified hydrogel as cardiac patches for cardiac tissue repair [49].

Carbon nanotubes are widely used in tissue regeneration for their morphological, physico-chemical, and mechanical properties, but mainly for their tunable conductivity depending on the CNTs nanostructure. The two following examples show how electrical, topographical, and mechanical properties of carbon nanomaterials can stimulate MSC differentiation or CMs adhesion and proliferation on nano-modified cardiac patches.

Electrospun scaffolds of PCL with CNTs were developed to promote the in vitro cardiac differentiation of human MSCs. An improved human MSC cardiomyogenic differentiation was obtained due to intrinsic electrical stimulation of CNT in the presence of 5-azacytidine [50].

The incorporation of carbon NFs into a scaffold of PLGA was used to realize a nano-reinforced cardiac patch where CMs showed an increased ability to adhere and proliferate. The effect of carbon NF nanotopographical features increased protein adsorption capacitance, thus forming a composite resembling native heart tissue [51].

#### 3.2.2. Inorganic-Organic Nanoparticles

Specific combinations of inorganic (i.e., AuNPs) and polymeric nanoparticles, allow the realization of a unique complex nano-system having a large repertoire of properties including the possibility of releasing, in a controlled way, therapeutic agents able to locally treat cardiac disease.

The use of synthetic polymers (i.e., aliphatic polyesters, polyethylene glycol) and biological polymers (i.e., chitosan, alginate, gellan) offers important advantages, higher than inorganic components, in terms of biocompatibility, biodegradability, ease of processing, and functionalization with many drugs or growth factors.

Polymeric nanoparticles can be obtained by a series of methods including emulsification, solvent evaporation, precipitation polymerization. Recently, curcumin capped AuNPs on PLGA were prepared by double emulsion–solvent evaporation method, these nanosystems demonstrated an efficient curcumin drug carrier for improved cardiac anti-hypertrophy activity in a rat model of induced cardiac hypertrophy [52].

#### 3.2.3. Polymeric Nanofibers

Non-biodegradable polymers, like polyacrylonitrile, can be processed by electrospinning to obtain aligned-orientated NF electrospun patch. This NF patch was used as a 3D scaffold to align the injected rat neonatal CMs and EC [62]. In vivo testing demonstrated that the cell-seeded cardiac patch after implantation after two months post-MI showed a reduction in infarct size and fibrosis, and improvement in cardiac function and cardiac haemodynamic parameters.

NF patches of cellulose fabricated via electrospinning were modified with chitosan/silk fibroin (CS/SF) multilayers obtained via layer-by-layer coating. The patches engineered with adipose tissue-derived mesenchymal stem cells were applied to the epicardium of the infarcted region in rat hearts. These patches demonstrated to promote the functional survival of engrafted SCs and reducing ventricular remodeling post-AMI through attenuating myocardial fibrosis [53].

PCL nanotubes obtained using electrospinning were used to fabricate porous patch for human MSC differentiation. In addition to induce electroconductivity PCL patch were modified with CNT.

Nanoscale design was applied to many synthetic biomaterials to closely mimic the ECM. For example, NF poly(L-lactic acid) (PLLA) matrix support cardiovascular progenitor cells growth and differentiation in 3D for cardiac tissue formation [54]. PCL and poly glycolic acid (PGA) NF scaffold with various PCL/PGA compositions. These scaffolds facilitated cell attachment, extension, and differentiation in vitro, and supported living cells expressing the key cell marker proteins of cardiomyocytes, smooth muscle cells and ECs in a mouse subcutaneous implantation model [55].

In general, synthetic or biological polymeric biomaterials, in the form of cardiac patches, can help cells to organize into functional tissue but, due to their intrinsic poor conductivity, limit the ability of the patch to contract. For this, a polymeric patch has to be reinforced or embedded with conductive nanomaterials using different methods to transform it in a conductive material [63].

There are some important electroactive polymers, such as polypyrrole (PPy), polyaniline (PANI), and polythiophene (PTh), which have demonstrated their potential as scaffolds and patches in cardiac tissue engineering. However, there are some important questions that need to be better investigated regarding mainly the biocompatibility, biodegradability, and capability of these biomaterials to be modified. Therefore, the use of electrically conductive nanostructured materials in cardiac tissue engineering requires more investigation involving an interdisciplinary approach [64].

Importantly, the use of polymeric nanomaterials to modify cardiac patch has to be considered, not for their possible electroconductive function but for their importance as drug delivery systems to treat myocardial ischaemia. A recent study demonstrated that NPs, based on deblock copolymers of PEG and poly (propylene sulfide) (PPS), loaded with ginsenoside Rg3, after intramyocardial injection in rat ischaemia-reperfusion model, improved the cardiac function and reduced the infarct size due to released Rg3 action in inhibiting oxidative stress, inflammation, and fibrosis [65].

An effective and multifunctional approach to treat myocardial ischaemia-reperfusion was developed by introducing relevant doses of a cardioprotective drug (adenosine) into multi-layered scaffolds having cardio-inductive properties on stem cells [66]. The further encapsulation of adenosine-core shell polymeric NPs into polymeric scaffold was implemented for a targeted controlled release of cardioprotective drug to improve the reperfusion injury treatment [67]. Intelligent polymeric NPs able to recognize a specific ECM metalloproteinase were also evaluated to modify cardiac scaffolds with the aim to prevent left ventricular dysfunction after AMI [68].

## 4. Cardiac Stem, Precursor and Differentiated Cell Interactions with Nanoengineered Materials

The evidence that SC interaction with nanoscale surface topography induced cell behaviour modification, made this interaction to become the principal topics in the future for both nanotechnology and SC research. Thus, tissue regeneration and function may be properly influenced by scaffold with nanoscale surface topography.

**Figure 3:**
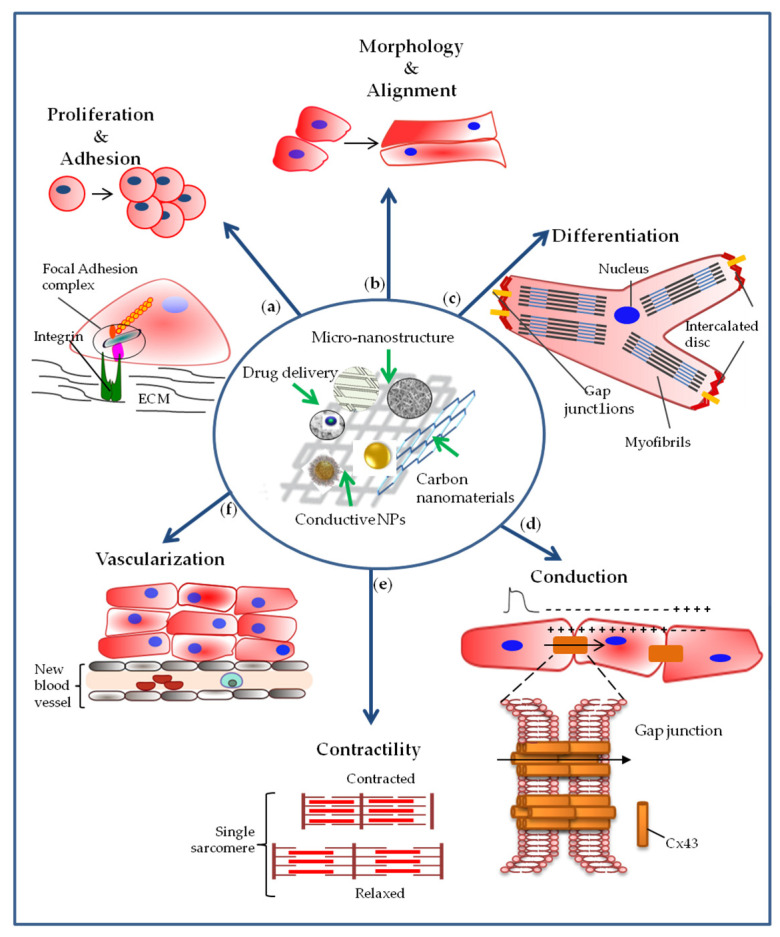
Representation of cellular effects required for myocardial regeneration, mediated by the interaction of stem cells and cardiac precursors with nanoengineered scaffolds. (**a**) The nanoengineered scaffolds can induce an increase in cell proliferation and adhesion; (**b**) they can also influence cell morphology by directing cells towards greater alignment, and (**c**) stimulate cell differentiation toward cardiac lineage with the organization of sarcomeric proteins in sarcomeres; (**d**) they can increase the expression of connexin-43 involved in the intercellular junctions with effects on cellular electrical coupling and on impulse conduction. (**e**) Cell-cell communication mediated by gap junctions together with cell differentiation also allow cardiac tissue to contract. (**f**) The use of nanoengineered scaffolds must also stimulate the formation of new blood vessels since the supply of oxygen and nutrients are essential for both viability and function of the new cardiac tissue.

The addition of nanomaterials can modify the physical and biological properties of the scaffold. Thus, different hybrid scaffolds containing various types of nanomaterials, with mechanical and/or electroconductive properties, have been created aimed at studying their effect on structural organization and functionality of various types of SCs and precursors (Figure 3).

### 4.1. Effect of Nanoengineered Materials on Cell Adhesion and Proliferation

A primary role in regulating cellular proliferation and adhesion has been attributed to the architecture at nanometre scale (Figure 3a). In particular, Bauer et al. pointed out that the cell response depends on the size of nanotubes. In fact, these authors reported that the greatest MSCs activity was observed on the layers formed by TiO_2_ nanotube with 15 nm in diameter. Moreover, comparing different coating of nanotube surface, the authors concluded that nanoscale surface topography of materials dominates over surface chemistry effects on MSC adhesion and proliferation [69]. Moreover, the proliferation rate of both MSCs and ECs decrease with the increase of TiO_2_ nanotube size [69,70]. In fact, after 72 h, MSC numbers strongly increased on 15 nm compared to 100 nm nanotubes diameter [69]. In another study, when cultured on anisotropic nanopatterned patch, the proliferation of multipotent cardiac progenitor cells (CPCs) increased, as confirmed by an increase in the expression of the marker of proliferation Ki67 [71]. Despite several works which reported that nanotopographical features of the matrix increases cell proliferation, most of the studies simply observed the enhancement in proliferation without explaining neither the predominant feature required nor the signalling pathway leading to this biological effect.

Since it is well known that cell adhesion is weaker on soft substrate compared to stiffer ones [72] the nanostructured PEG hydrogel determines weak increase in neonatal CMs adhesion to the substrate, despite non-adhesive nature of bare PEG hydrogels. The nanopillar topography of PEG favoured cell–cell interactions with consequent cell aggregation and growth. In fact, the softer surface of nanopillar PEG promotes growth compared to the stiffer surface of bare PEG that promotes migration [73].

Culture of CPCs on NF scaffolds made up of PCL and PGA with various proportion evidenced the highest cell adhesion and proliferation on the synthetic scaffold with 65:35 PCL:PGA ratio, ascribing this effect to the enhanced hydrophilic properties. Whereas an increase of more than 50% in PGA showed a gradual reduction in cell adhesion and proliferation because of hydrophobic feature [55]. Another study on scaffolds made up by different ratios of PLGA and CNF highlighted that changes in composition can drive cell differentiation towards different tissues [74].

On the other hand, Wickman et al. demonstrated that the adhesion of CPCs was affected by the structural characteristics of the patches rather than by their composition. Indeed, when PCL was fabricated as sheets there was no CPC adhesion even if the PCL was integrate with thiophene-conjugated CNT, while when PCL was produced as fibrous meshes by electrospinning there was adhesion and proliferation of CPC [75]. It should be underlined that PCL sheet alone is a hydrophobic polymer with little biological activity and the addition of thiophene-conjugated CNT did not improve it. This flat sheet did not allow the retention of the cells, while the 3D scaffold obtained by fibrous meshes made by PCL increased it. Moreover, adhesion and retention of the cells were more marked with the addition of thiophene-conjugated CNT. However, CNT-PCL favoured CPC proliferation with maintenance of immature phenotype as receptor tyrosine kinase (c-kit) and insulin gene enhancer protein 1 (Isl-1) expression was demonstrated [75]. CNT-PCL composite scaffolds increased also the adhesion of human MSCs [50].

To improve the effectiveness of the nanomaterials, the mechanical properties were integrated with the electrical one. The addition of CNT into Gelatin-Methacryloyl (GelMA) hydrogel promoted adhesion, retention, and viability of neonatal CMs which were not observed on pristine one [56]. A strong cells adhesion has been observed with graphene oxide (GO)-GelMA compared to GelMA alone [76]. Furthermore, these authors observed a rise in SC adhesion when GO was partially reduced. An additional enhancement of cell adhesive properties was also observed when GO was coated with a natural polypeptide poly L-lysine (PLL). Nanoparticle shape affected focal adhesions number and size thus determining cell adhesion strength [77]. However, it must be taken into account that each component added to the scaffold can affect its properties. For instance, the addition of PLL to GO reduced the conductance of GO.

Also, gold nanorod (NR)-embedded hydrogels induced an increase in stiffness of the scaffold leading to an enhancement in neonatal CMs adhesion, with formation of tissue layer integrated with the scaffold [78]. Comparing the addition of various concentrations of AuNR to GelMA, cell retention and adhesion increased with the highest concentrations. The authors asserted that matrix stiffness influences cell behaviour, but the conductive component exerted a pivotal role in improving cell adhesion and retention. Higher AuNR concentration in the hybrid construct induced an enhancement in β1-integrin expression that favoured their ability to adhere, thus confirming cell–matrix interaction is mainly mediated by β1-integrins.

The nanotopography of biomaterials seems to be the most important cue for cell adhesion and proliferation. Moreover, the control of some other features of the nanomaterial such as stiffness, hydrophilicity and anisotropy can still influence (improve or lessen) the interaction between cells and nanomaterial surface. With a few exceptions, the addition of conductive component to the nanostructured matrix can enhance even more the ability to adhere, likely increasing the integrins that usually mediate cell adhesion.

The enhanced adhesion of neonatal CMs on CNT-integrated collagen scaffold was accompanied by an increased expression of disc-related protein with consequent enhanced intercalated disc assembly and functionality among the CMs. This property may be important for the integration with the host tissue [57]. It is already known that intercalated discs are composed by three different components: adherens junctions are specialized in the transfer of contractile forces between cells, desmosomes are specialized in cell-to-cell adhesion and gap junction are specialized in the transfer of impulse between neighbouring cells. The authors unveiled the pathway that triggers the increased formation of these intercellular structures that are a feature of myocardial tissue. In fact, they showed a pivotal role of β1-integrin-mediated RhoA signaling pathway in the formation of mechanical junction such as adherens junctions and desmosomes were attributed to [79].

### 4.2. Effect of Nanoengineered Materials on Cell Morphology

Initially, the alignment and elongation of the neonatal CMs within the 3D hydrogel scaffold was induced with electrical and/or mechanical external stimulation [12,14,80,81]. More recently, it was pointed out that alignment of NFs mimics the anisotropic structural organization of myocardium [82] and the addition of well orientated nanostructures, such as nanofibers or nanotubes, in 3D scaffold allowed to reach the alignment and elongation of the cells without external stimuli (Figure 3b). In fact, 3D patches with PLGA-NF alignment, that mimics the decellularized ECM of rat cardiac tissue, showed that human iPSC-CMs cultured on it are aligned with the same direction of NFs in contrast to the random distribution of iPSC-CMs in 2D culture [83]. An alignment was also observed in neonatal CM cultured on alginate NW scaffold, while rounded shape cells formed aggregates when cultured on bare alginate [46]. When cultured on CNT-collagen hydrogel [57] as well as on CNT-GelMA [77], neonatal CMs showed enhanced cell elongation and morphology alignment leading to a well-organized tissue formation. Another study was focused on the orientation of the neonatal CMs by the assessment of the angle between cellular long axis and the direction of aligned NFs inside a 3D GelMA scaffold. It is relevant that the alignment and the elongated morphology of the cells was still maintained after 6–12 h of enzyme (lipase) activity which disrupted the nanoscale component (only micrometre-scale was observed by SEM) [84]. The acquired cellular distribution and morphology can favour cell–cell coupling that is fundamental for tissue engineered functionality.

Native myocardium is composed by multiple layers. A similar structure can be achieved in highly organized 3D construct using the layer-by-layer assembly technique, that consists in the deposition on cell surfaces of nanometre-thick films which works as an inter-layer spacer for the layer-by-layer assembly [77]. Nano-films can display different physical and biological properties depending on the components.

It must be taken into account that in each layers CMs are uniformly aligned in native myocardium, while there is a gradual transition in orientation of the aligned cells among the layers. On the basis of these facts, a precise orientation is required for suitable mechanical properties in cardiac regeneration. Wu et al. claimed that a 3D multilayer construct with properly differently orientated cells could be beneficial for cardiac regeneration. In fact, they attempted to overcome this limitation trying to reproduce the structure of the native cardiac tissue. Since the authors could control cellular orientation, they realized a 3D construct assembling two monolayers, with orthogonal cellular orientation with respect to each other, previously encapsulated into a GelMA hydrogel shell [84]. This work demonstrates the great potential of anisotropic nanostructures. However, in regenerative medicine, the limitation of an engineered multi-layered 3D tissue is its insertion in the cardiac tissue, because it is difficult for the orientation of each layer of the 3D construct to precisely reflect the orientation of the native cardiac cells.

### 4.3. Effect of Nanoengineered Materials on Cell Differentiation

The nanocomposite hydrogels obtained by CNT incorporation into collagen, provided an instructive extracellular microenvironment that favoured brown adipose-derived stem cells (BADSCs) proliferation and differentiation as demonstrated by the expression of cardiac transcription factors GATA-4 and Nkx2.5 as well as sarcomeric proteins: cTn T, α- and β-myosin heavy chain (α-MHC, β-MHC), and α-SA, at day 21 [85]. Also, embryoid bodies increased their cardiac differentiation (i.e., increased expression of Tn T as well as of the cardiac genes Tnnt2, Nkx2-5, and Actc1) when cultured in the microwells fabricated on a scaffold obtained by the addition of CNT to GelMA [25]. CNT-GelMA hydrogel promoted maturation of neonatal CMs, which was not observed on pristine one [56]. After two weeks, iPSCs cultured on gelatin-coated 3D PCL-NF scaffold spontaneously differentiated, without any exogenous stimulation, towards cardiac lineage as demonstrated by the increase in cardiac marker expression such as cTn T, cardiac myosin light chain (MLC) and α-SA. The authors highlighted that, in the early phase (d0-3), this differentiation process was mediated by Wnt/β-catenin signalling pathway [17]. On the other hand, Sun et al. suggested that the cardiac differentiation of BADSCs was due to a β1-integrin-mediated transforming growth factor-β1 (TGF-β1) signaling pathway [85]. Nanomaterial-induced differentiation of various cellular types towards cardiac lineage [17,24,46,69,75,77,85] via the expression of specific proteins which allow stem cells to acquire a morphology and function typical of the mature phenotype (Figure 3c).

Lee et al. compared the effect of GelMA hydrogels embedded with three different carbon-based NPs. In particular, GO and reduced GO showed similar shape but varied in surface chemistry and conductivity while CNT and reduced GO have comparable conductivity but showed different shapes and surface area. An electrophysiology study (after patch-clamp) pointed out a difference on neonatal CM phenotype and maturity among the three different substrates. It is likely that different compositions of the three substrates create various microenvironments which trigger a different neonatal CM response. In fact, only the neonatal CMs seeded on CNT-GelMA assumed a ventricular-like phenotype. The GO-GelMA induced an atrial-like phenotype, while reduced GO-GelMA generated a mixture of the two phenotypes [86]. We can state that this study clearly highlighted that conductive materials affect the cell behaviour and play an important role in cell fate.

This study pointed out that although the electroconductive materials favoured the differentiation towards cardiac lineage, the chosen substratum could strongly influence the result. These results seem to indicate that CNT-GelMA is more suitable for cardioregeneration, since after myocardial infarction usually a ventricular damage occurs. Moreover, this study underlines the importance of a deeper functional investigation that could add more information on the phenotype of the cells in order to achieve the goal of cardioregeneration.

Several authors have shown an initial organization of sarcomeric protein in sarcomeres. Neonatal CMs cultured on CNT-GelMA exhibited a remarkable striation of F-actin [76]. CNT-PCL composite scaffolds increased differentiation of human MSCs, indeed in these elongated cells the sarcomeric protein MHC and F-actin are strongly colocalized [50]. In another study, neonatal CMs cultured on CNT-loaded collagen hydrogels showed sarcomeric structures with well-defined anisotropic and isotropic bands and massive actinin striation compare to collagen hydrogel alone [79]. When cultured on aligned NFs-embedded GelMA scaffold, neonatal CMs acquired clearly orientated α-SA and showed increased sarcomere length compared to the control [84]. The electric conductivity of GO in MSCs induced a gene expression increase of early marker GATA-4 as well as of the contractile proteins (i.e., MHC, MLC, and cTn T). While GATA-4 decreased with the culture time, a further up-regulation of sarcomeric proteins was present at week 3. However, MSC cultures on double- and triple-layer graphene did not exhibit an additional increase in cardiomyogenic gene expression. Since MSCs did not exhibit the functional and electrophysiological properties of mature CMs regardless of the substrate type, the authors suggested that additional signals may be required to differentiate MSCs into mature CMs, like graphene [87]. Thus, it seems that the addition of electroconductive nanomaterials induced also a more pronounced differentiation towards cardiac lineage of various cellular types cultured on them, though it should be kept in mind that the degree of differentiation achieved depended also on the type of SCs involved in the study. In fact, the increased expression in cardiac markers matched with the enhanced level of Cx43, a membrane protein involved in cell–cell coupling, as evidenced in the next subsection.

### 4.4. Effect of Nanoengineered Materials on Cellular Electrical Coupling and Conduction of the Impulse

Connexins are proteins involved in the intercellular junctions called gap junction. In fact, gap junctions are formed by the proper docking of two connexons that in turn are composed by hexameric assemblies of connexins. Among the different isoforms, the main one is Cx43. These structures, localized at the intercalated disc of the CMs, allow the passage of ions form one CM to the next one.

After cell isolation, Cx43 is lost or internalized into the cell, thus limiting the electrical coupling with adjacent cells and consequently the proper cell–cell transfer of the electrical signal, function that is essential in the cardiac tissue.

To achieve the highly beneficial effect of electrical property enhancement, which is fundamental in cardiac applications, AuNPs were deposited in decellularized scaffolds composed of a fibrous matrix. Neonatal CMs seeded on this scaffold showed up an increase in Cx43 [88,89]. The expression of Cx43 was also increased in MSCs cultured on GO [87].

The cellular distribution of this protein is most relevant for an adequate transmission of electrical signal between cells, thus improving engineered tissues function suitable in MI treatment. Cells cultured on various AuNP scaffolds showed that Cx43 is especially localized aligned between adjacent neonatal CMs while its expression is weaker and randomly distributed in cells cultured on pristine scaffold [88,90]. The distribution of Cx43 in membrane is essential for a synchronized electrical signal propagation, typical of cardiac tissue. Incorporation of AuNWs in alginate scaffolds augmented electrical properties of the scaffold and facilitated transmission of the electrical signal. When neonatal CMs interacted with NWs-integrated scaffold, the engineered tissue showed a faster and synchronized electrical activity [46,56]. On the contrary, pristine scaffold showed negligible signal conduction among cells, even after external stimulation [46]. On the other hand, alginate films containing AuNRs, instead of NWs, exhibit similar topographic features, but a negligible electrical current was measured [46,56]. All together these results demonstrate an essential role of electroconductive materials in the signal propagation, accompanied by an increased expression as well as by a change in distribution of Cx43 (Figure 3d).

Other groups observed spontaneous electrical activity in neonatal CMs grew on CNT-laden scaffold, with a consequent cell–cell coupling improvement [56,91]. Martinelli et al. highlighted that the addition of CNT to the scaffold changed the electrophysiology of the cells. In particular, neonatal CMs cultured on CNT-scaffold exhibited a more negative resting potential that was closer to that of mature CMs [91] and the authors attributed this cell maturation to the interaction of neonatal CMs with CNT.

Using AuNWs as conductive materials within 3D porous scaffolds, neonatal CMs exhibited enhanced electrical coupling and later on growth of cardiac muscle cells was observed and resulted in electrical synapse formation [58].

Shun et al. studied the signaling cascade that brings to the enhancement in Cx43. The authors demonstrated that the formation of gap junction occurred via the activation of the β1-integrin-mediated focal adhesion kinase - extracellular signal-regulated kinase - GATA-4 transcription factor (FAK/ERK/GATA-4). This pathway triggered by β1-integrin should be studied with different conductive materials to confirm its role. Therefore, conductive nanomaterials notably hasten gap junction formation, thus providing the cues for the spreading of the impulse into the cardiac tissue, a key activity in cardiac function [85].

### 4.5. Effect of Nanoengineered Materials on Cell Contractility

Effective cell–cell communication among adjacent cells is important for cardiac tissue contraction. It is well known that myocardial function is a consequence of electro-mechanical coupling that allows to convert the cellular electrical excitation in mechanical contraction. In fact, the propagation of excitation (i.e., action potential) through gap junction to neighbouring cells cause an increase in cytosolic Ca^2+^ level. The binding of Ca^2^ to cTn C activates the sliding of sarcomeric proteins that lead to cell contraction. Yet, a proper correlation exists between the structure and the function of the myocardium, indeed the cardiac fibres can twitch only if the sarcomeric proteins give rise to sarcomere structures (Figure 3e).

Since the myocardium must contract simultaneously, i.e., as a syncytium, it is essential that the contraction is preceded by the spreading of impulse throughout the gap junctions. An enhancement of intercellular connection between aligned neonatal CMs, due to the increase in Cx43 expression, together with the improvement of sarcomere structure, are essential for the contractile property of the heart. Indeed, along with a higher expression of cardiac markers (such as cTn I, α-SA, and Cx43), neonatal CMs cultured on alginate NW exhibited a synchronous contraction after stimulation [46] and synchronous spontaneous beating, that lasted for more than a week, was shown when the neonatal CMs were seeded on CNT-laden GelMA [56]. Instead, BASCs cultured CNT-PCL scaffolds beat spontaneously for up to three days only [85]. Shin et al. attributed the more stable synchronous beating of the cells to a lower excitation threshold [56]. A reduced excitation threshold was observed in 124 polymers integrated with CNT [92]. Conversely, the synchronous contraction of the cardiac cells seeded on AuNP-decellularized matrix occurred applying a lower voltage with an external electrical pacing [93]. Therefore, the reduction of excitation threshold is not just a feature of CNT.

On the contrary, neonatal CMs cultured on nanopillar PEG generated train of action potentials with higher amplitude and different morphology compared to control. Surprisingly, beating frequency was lower than expected: it was similar to the one measured in the bare PEG. The author suggested that the neonatal CMs beating might be affected by insufficient network formation and/or weak cell–cell adhesion as well as poor interaction with the substrate [73]. It is likely that an improvement in the effectiveness of the nanomaterials could be obtained if the mechanical properties were integrated with the electrical one.

Electroconductive nanomaterials enhanced contractility. Changes in calcium level has been observed in the case of neonatal CMs cultured on AuNR-GelMA hybrid hydrogel [78]. In particular, a higher efficiency in calcium cycling has been observed in iPSC due to the presence of aligned NFs in 3D scaffold [83]. Moreover, calcium transient is essential for the rhythmic synchronous beating of the cells. Calcium transients were significantly enhanced in neonatal CMs cultured on NWs-loaded alginate than in alginate alone [46]. Using a green fluorescence dye, the authors demonstrated that applying an external stimulus to the pristine scaffold the fluorescence remained localized in the stimulation point, while in the NWs integrated scaffold the propagation of the depolarization occurred and reached remote areas from the stimulation point [46]. Spontaneous Ca^2+^ transients with higher fluctuation and more synchronous rhythm were observed in neonatal CMs cultured on CNT-collagen hydrogel compared to bare collagen [57]. The enhanced contractility occurred also in neonatal CMs cultured on cardiac patches formed by CNT-incorporated chitosan-gelatin hydrogels [94]. Interestingly, beating propagation can be interrupted by heptanol that inhibits the calcium permeability of gap junction thus inhibiting cell–cell coupling. Beating persisted in cells cultured on CNT-GelMA compared to the GelMA alone. This persistent activity of the cells in the presence of the heptanol was attributed to the conductive CNT network which allowed the transmission of action potential between cells [56].

All together these results suggest an important role of electroconductive nanomaterials as 3D scaffold components, because their conductive property could be helpful in an infarcted area of the myocardium where necrotic CMs interrupt the propagation of the action potential in the ventricular tissue, thereby increasing the probability that arrhythmic events occur.

Greater isometric twitch forces were observed in tissue-engineered cardiac patches with a remarkable degree of CM alignment and electrical anisotropy [90]. It should be kept in mind that, in native myocardium, the precise orientation of the CMs is essential for an efficient twitch of the heart during the contraction. In fact, seeded iPSC-CM on decellularized thin slices of myocardium, obtained from mini pig hearts with a native alteration in ECM due to genetic hypertrophic cardiomyopathy, displayed prolonged twitch contractions (due to a prolonged calcium transient) [95]. These authors highlighted the pivotal role of ECM architecture in tissue engineering, demonstrating how alteration in myocardial ECM can also alter the contractile behaviour of healthy cells.

### 4.6. Effect of Nanoengineered Materials on Vascularization

Incomplete vascularisation and poor endothelialization can limit the potentialities of engineered cardiac scaffolds for regenerative purposes after tissue damage.

It is well-known that blood vessel wall is formed by three different layers: intima, media, and adventitia, starting from the innermost outwards. Although each layer included at least one precursor population which can be involved in new vessel formation, it has been reported that growth factors exerted a relevant role in this process [96] As stated earlier, vascularization is essential to guarantee the vitality of cardiac tissue and to maintain its function since CMs have high metabolic request. Moreover, about 30% of cardiac tissue is composed of myocytes, while the remaining 70% are non-myocytic cells (such as fibroblasts, endothelial and smooth muscle cells) that support the growth of myocytes [97,98]. Engineering multilayer constructs display some limits. In particular, nutrients and oxygen transport to the cells in the deepest layers are restricted because of the thickness of the in vitro engineered tissue [99]. It has been reported that in a construct formed by more than three sheets (80 μm thick) an extensive necrosis occurred [100]. Vascularization of cardiac engineered multilayer tissue could overcome this critical limit, thus promoting the long-term survival of the artificial tissue, which is a highly relevant for clinical application in cardiac regeneration (Figure 3f).

It should be born in mind that coronary circulation supports myocardial development not only via perfusion, but also through paracrine release of various factors/molecules [101]. It is established that blood vessel formation can occur by (1) angiogenesis which is the sprouting of new blood vessels from the existing ones and endothelial cells are responsible for the capillary growth, (2) arteriogenesis that consists of remodelling and growth of pre-existing arterioles for instance by the increase in their lumen size or (3) vasculogenesis that consists of de novo vessel formation involving the migration, differentiation and incorporation of endothelial progenitors, normally from the bone marrow, into the damaged vessels. During ischemia neovascularisation is favoured by autocrine factor release which activate endothelial cells to respond to exogenous growth factors, in particular vascular endothelial growth factor (VEGF), fibroblast growth factor (FGF), insulin growth factor (IGF) [102].

Several studies focused their attention on vascularization of constructs, via the functionalization of NPs with growing factors to induce the formation of vessel network. Tan et al. produced self-assembled NPs capable of sustained VEGF release to augment vascularization of decellularized scaffolds obtained from bovine jugular vein [103]. Non-covalent self-assembly properties of heparin with low molecular weight and N,N,N-trimethylchitosan chloride (TMC) through electrostatic interactions allowed to obtain NPs able to protect VEGF bioactivity, demonstrating both higher proliferation of ECs and increased capillary formation rate. In in vitro study by Gonzalez-García group demonstrated that poly(ethyl acrylate) triggers the assembling of fibronectin into nanonetworks, fibronectin remains in a globular conformation within the control polymer poly(methyl acrylate). The fibronectin network allows VEGF binding domains to be exposed, thus promoting vessel formation by endothelial cells in response to extremely low concentration of VEGF inside the scaffold [104]. Izadifar et al. designed bilayer polymeric NPs made of different components (PLGA and PLLA/PLGA shell/cored bilayers) in order to obtain sequential release of angiogenesis factors from a fibrin matrix, platelet-derived growth factor first followed by simultaneous release of basic FGF and VEGF [105]. Rat aortic ring assay was used to assess ex vivo angiogenesis and the highest significant rise in the number of endothelial sprouts was obtained in sequential release group in comparison to simultaneous VEGF-FGF and only VEGF release groups. In another study, angiogenic growth factors were encapsulated into NF scaffolds, trying to mimic the complexity of native cardiac ECM architecture, which exhibits a NF-collagenous matrix with spatio-temporal localization of various growth factors. Lakshmanan’s group highlighted the efficiency of growth factors embedded NF patch made of two polymers, poly(L-lactide-co-caprolactone) (PLCL) and poly(2-ethyl-2-oxazoline) (PEOz) to stimulate EC migration and vasculogenesis in in vitro study. Moreover, the implant of this dual growth factor-loaded patch in a rabbit having undergone AMI evidenced blood vessel sprouting in the patch secured region with consequent improvement of functional regeneration of cardiac tissue [106]. He et al. confirmed that neovascularization was accompanied by a reduction in infarct size extension as well as cardiac function improvement in an in vivo study [107].

Since the addition of ECs to CM culture exerted a positive effect on the spontaneous vascularization, improving cardiac tissue structure and function [101,108,109], other authors developed strategies to obtain engineered endothelialized cardiac tissues via ECs and CMs coculture on 3D scaffolds. In particular, adding GelMA shell encapsulating homogeneously ECs network to a single layer 3D scaffold covered by elongated and aligned CMs, an in vitro vascularized tissue surrogate was obtained [84]. The interaction of ECs with CMs may also promote cell viability and proliferation [101].

Nevertheless, considering the structural and functional hierarchy of coronary circulation of the native myocardium, neovascularization and its integration in the host tissue remains a great challenge in tissue engineering.

More attention, however, should be paid to the choice of nanomaterials because some negative effects have also been reported. For example, diamond NPs and, to a lesser degree, graphite NPs inhibited angiogenesis [110]. Moreover, TiO_2_ nanomaterials induced leakiness of blood vessels [111]. AuNPs also caused leakiness depending on the NP size and different sensitivity of endothelial cells according to their various origins [112].

## 5. Challenges and Future Perspectives

Tissue regeneration and function may be properly influenced by scaffolds with nanoscale surface topography. For about three decades, researchers involved in cardiac tissue engineering have been evaluating the possibility of adapting and implementing several micro and nanofabrication techniques to produce 3D nanostructured biomaterials in order to provide a native-like template of ECM that stimulates a proper SC commitment towards cardiac lineage. Indeed, the improvement and/or acquirement of some biological properties such as proliferation, adhesion, differentiation, mechanical properties, electrical properties, and vascularisation could be achieved by combining different nanomaterials, thus taking advantage of their different properties and features. The use of hybrid materials, based on the combination of organic (i.e., synthetic polymers, proteins, polysaccharides, and their blends) and inorganic components, provides improved and tunable mechanical and rheological properties, leading to a better performance of the scaffolds for soft tissue engineering.

These intense studies allowed to define a few important concepts: (i) the nanotopography of biomaterials seems to be the most important cue for cell adhesion; (ii) the addition of conductive component to the nanostructured matrix can enhance even more the ability of the cell to adhere and, certainly, to differentiate; (iii) the chosen conductive substratum can strongly influence the differentiation result and specific attention has to be deserved to the ventricular-like phenotype; (iv) conductive nanomaterials is a key component for gap formation and impulse conduction; (v) well-oriented nanostructure, that mimics the anisotropy of ECM, affects elongation and alignment of the cells which is crucial for well-organized tissue formation; (vi) beside regeneration, multiple properties of the nanomaterials, like stiffness, elastic modulus, interfacial adhesion, and particle size exert an essential and prompt mechanical support for the infarcted myocardium.

The nanomaterials (nanoparticles, nanotubes, or nanofibers) studied in the literature and reported in this review have shown to have important properties for cardiac tissue engineering, allowing the implementation of the functions performed by the scaffold alone. Each kind of nanomaterial can improve a particular function, even if it can sometimes elicit multiple actions, therefore the choice in its use depends on the main aspect to be improved. In particular, AuNP have been used for their high thermal conductivity, low toxicity, and capability of enhancing electrical communication between adjacent cardiac cells. Moreover, various strategies have been already developed to modify their surface in order to use these nanomaterials also as drug delivery nanocarriers for a controlled release of specific biomolecules capable of improving proliferation and differentiation of stem cells. The challenge remains to find a correct spatial distribution of conductive nanoparticles. With respect to this, nanofibers and nanotubes would favor a greater connection leading to a nano fibrous network that reduces the electrical impedance of cross-linkable hydrogels. Actually, the main reason for the use of nanofibers and carbon nanotubes is their efficiency in increasing mechanical strength of scaffold in order to withstand the high pressures of each heartbeat and volume of blood. The main limit in the use of metallic or inorganic nanoparticles is their non-biodegradability and possible chronic bioaccumulation with consequent toxic effects. The use of polymeric nanomaterials and, in particular biodegradable polymeric nanoparticles, could be beneficial in this sense but they do not possess conductive properties in order to favor cell differentiation, impulse propagation and cell contractility. To increase electrical conductivity, the use of electroactive polymers could be very promising, however the greatest challenge is the potential toxicity and non-degradability of these materials. If we consider the approach of heart tissue engineering in vivo, the limitations related to the use of permanent electroactive polymers are even stronger. For this, many efforts in multidisciplinary research have to be addressed to enhance the mechanical and electrical properties of the scaffold, while maintaining the main requirements of multifunctional scaffolds in terms of biocompatibility and biodegradability.

Many manufacturing techniques have proven to be suitable for the realization of micro-nanostructured scaffolds mimicking anisotropic structure of myocardium. However, each of these techniques presents some advantages and disadvantages. Laser-based techniques, 3D printing and electrospinning allowed the direct fabrication of 3D scaffolds having different spatial resolution, using biological and elastomer polymers also in the presence of cardiac progenitor cells, showing improved cardiac function after in vivo implantation. Moreover, a large number of materials including biological, synthetic and bioartificial polymeric materials were processed in micro-structured substrates to be subsequently overlayered to obtain a 3D patch. In recent years, one of the best and most applied techniques is electrospinning that offers the possibility of recreating the fibrous structures of cardiac ECM from the nano- to micro-scale. Obtained polymers in form of nanofibers can be assembled in different forms and implemented with electro-conductive nanomaterials and biochemical signals to provide the mechanical, conductivity and biological properties of cardiac microenvironment. This technique consents the control of fiber diameter and alignment, but presents some limitations in the formation of thicker constructs and consequently in mechanical strength. Recent advances in fabrication of nanofibers using novel apparatus of electrospinning system could bring addressing specific issues, in order to manufacturing high- quality 3D scaffolds [113]. Additive manufacturing, including selective laser sintering and bioprinting, consents to produce a 3D construct on the basis of a predefined CAD drawing. The resolution depends on the physico-chemical properties of materials used, laser size and viscosity in the case of hydrogel. However, for some materials, heat generation can cause deformation of structure profiles or alteration of chemical properties of patch. For this reason, advances in combinatorial approach of microfabrication techniques can reveal particularly useful to meet the challenges for a valid in vitro but particularly in vivo cardiac tissue engineering.

In addition, 3D printing of cell-laden soft hydrogels can be considered an emerging strategy to recreate the native micro-environment by means of a specific composition and positioning of cells and materials in one step. A 3D printed pre-vascularized stem cell patch was produced using stem cell-laden decellularized ECM bioinks showing improved cardiac functions as well as reduced fibrosis and capillary formation [114]. New multicomponent material bioinks including nanoparticles or inorganic fillers are currently being developed to improve the mechanical resistance and stiffness of pure hydrogels, to increase the printing fidelity and to elicit desired cell functions for specific tissue engineering applications [115]. Composite bioinks have already demonstrated great potential for hard tissue engineering but due to their tunable and personalized properties have been gaining increasing attention also for soft tissue engineering.

From a biological standpoint, new insight about the pathways triggered by the interaction of the cells with the mechanical, structural, and electrical properties of nanomaterials would be helpful to achieve successful myocardial regeneration. Unfortunately, most studies described just the appearance of biomolecular or morphological/histological cardiac features, such as the appearance of sarcomeric proteins or the expression of transcription factors typical of cardiac cells. To date, however, few studies have revealed the molecular pathways triggered by nanomaterials that cause changes in cellular behaviour, with the possible exception of the increased cellular adhesion and cell–cell interaction. The knowledge of pathways involved in other nanomaterial-induced cellular effects, a deeper investigation on the functional aspects of the 3D engineered cardiac tissue, as well as a definition of the precise spatial and temporal control exerted by nanomaterials over the physiological phenomena through real-time monitoring of relevant biological events are very important future challenges.

## Figures and Tables

**Table 1:** Representative examples of scaffold materials, cells and micro-nanofabrication techniques for myocardial tissue engineering (MTE) with their relevant biological effects.

Material	NM	Cells	Approaches/Method of Assembly	Specific Application	Biological Effects	Ref.
GelMA and MeTro		neonatal rat CMs	Micromolding, photolithography	in vitro MTE	Cell attachment and beating	[28]
PEG		CMs	Soft lithography	in vitro MTE	Alignment of the focal adhesions	[29]
PS		Human AM-MSCs + mouse ESCs	Soft lithography	in vitro MTE	Early differentiation of mESCs (in cardiac-like beating cells) and heterogeneous cells	[30]
Agarose		C2C12	Laser ablation	in vitro MTE	Improved attachment and differentiation of myoblasts	[31]
PGS		neonatal rat CMs	Laser-assisted technique	in vitro MTE	Increased alignment of heart cells and improved mechanical properties	[32,33]
PHB		MSCs, CMs, CFs	Electrospinning	in vitro MTE	Induced angiogenesis, reparative process and remodeling	[34]
PLGA		neonatal rat CMs	Plasma etching, adsorption fibronectin	in vitro MTE	Modulation of gene expression in cells	[35]
BSA/PVA	AuNPs	Human MSCs	Electrospinning	in vitro MTE	Cardiogenic differentiation of MSCs	[36]
PMGI + heparin-binding peptide I	AuNPs	HeLa, hPSCs	Co-electrospinning	in vitro MTE	Enhanced HeLa cell attachment and potentiated CM differentiation of hPSCs	[37]
PGS/gelatin	CNTs	CMs	Electrospinning	in vitro MTE	Superior mechanical properties, enhanced CM beating properties	[38]
PLGA + YIGSR		neonatal rat CMs	Electrospinning	in vitro MTE	Higher expression of a myosin and b-tubulin, faster and latest longer contraction of CMs	[39]
PLGA + SF + Aloe Vera		CMs	Electrospinning	in vitro MTE	Increased cell proliferation and enhanced cardiac expression proteins	[40]
PCL	CNTs	H9c2	3D-printing	in vitro MTE	Cell proliferation	[41]
GelMA		hciPSC-CMs, -SMCs, -ECs	3D-MPE	in vitro MTE	Potentiated cell viability, electromechanical coupling in vitro, improvements in cardiac function, infarct size, vascularizing in a murine MI model	[42]
PEGDA-Wp in PEGDA hydrogel		Human CPCs	Microstereolithography	in vitro MTE	hCPCs differentiation and 3D spatial orientation, activation of connexin 43 expression	[43]
ECM/SF	AuNPs	MSCs, CMs	Spray drying machine	in vitro MTE	Higher cell survival and retention of CMs	[44]
cholecyst-ECM	AuNPs	H9c2	EDC-NHS functionalization to cholecyst -ECM	in vitro MTE	Suitability for the growth and proliferation of cardiomyoblasts	[45]
Alginate	AuNW	CMs and fibroblasts	Ionic crosslinking	in vitro MTE	Improved electrical connectivity and functionality	[46]
ECM	Lap-AuNPs	CMs	Decellularization process	in vitro MTE	Effective cardiac protein expressions and phenotype maturations of cardiac specific proteins on the CMs	[47]
Chitosan	Se-NPs	H9c2	Film preparation	in vitro MTE	Attachment and proliferation of H9c2 cells, functional electrical connectivity between heart cells and films	[48]
PEG/Chitosan	TiO_2_ NPs	neonatal rat CMs	Chemical crosslinking	in vitro MTE	Adhesion, elongation and spreading of cells	[49]
PCL + azacytidine	CNTs	Human MSCs	Electrospinning	in vitro MTE	In vitro cardiac differentiation of hMSCs	[50]
PLGA	CNF	Human CMs	Film preparation	in vitro MTE	Increased CM growth, increased conductivity of the composites	[51]
	CAu-PLGA NPs		Double emulsion solvent	in vivo drug delivery for cardiac anti-ipertrophy	Improved survival rate and in vivo cardiac ant-hypertrophy	[52]
Cellulose + CS/SF		AD-MSCs	Electrospinning	in vitro MTE	Reduced ventricular remodeling post-MI	[53]
PLLA		CPCs	Sugar template, freeze drying	in vitro MTE	Cell extension, growth and differentiation towards desired lineages in vitro, larger number of living cells	[54]
PCL:PGA		CPCs	Electrospinning	in vitro MTE	Cell attachment and differentiation in vitro and support living cells in vivo	[55]
GelMA	CNT	neonatal rat CMs	Hydrogel preparation, UV irradiation	in vitro MTE	Transmission of action potential between cells and synchronous spontaneous beating	[56]
Collagen	CNT	neonatal rat CMs	Hydrogel preparation	in vitro MTE	Spontaneous Ca^2+^ transients and synchronous rhythm	[57]
GelMA, PEG	CNT	neonatal rat CMs	Microelectrode array, photolitography	in vitro MTE	Enhanced electrical synapse formation and electrical coupling	[58]

NM–nanomaterials; PS–Polystyrene; PGS–Poly (Glycerol Sebacate); AM-MSCs–amniotic membrane-derived mesenchymal stem cells; ESCs–embryonic stem cells; PHB–Poly(3-hydroxybutyrate); MSCs–Mesenchymal stem cells; CMs–cardiomyocytes; CFs–Cardiac fibroblasts; PLGA–Poly(lactic-co-glycolic acid); BSA/PVA–Bovine Serum Albumin/Poly(vinyl alcohol); PMGI–Polymethylglutarimide, CNT–Carbon nanotube; YIGSR–Tyr–Ile–Gly–Ser–Arg; SF–Silk Fibroin; PCL–Poly(ε-caprolactone); GelMA–Gelatin-Methacryloyl; SMCs–smooth muscle cells; ECs–Endothelial cells; MPE–multiphoton-excited; PEGDA-Wp–Poly(ethylene glycol) diacrylate woodpile; EDC-NHS–1-ethyl-3-(3-dimethyl aminopropyl)-carbodiimide/N-hydroxysuccinimide; PLLA–Poly(L-lactic acid).

## Abbreviations

3D	Tridimensional
AMI	Acute myocardial infarction
BADSC	Brown adipose-derived stem cell
CM	Cardiomyocyte
CNT	Carbon nanotube
CPCs	Cardiac progenitor cells
CS/FS	Chitosan/silk fibroin
cTn	Cardiac troponin
Cx43	Connexin 43
EC	Endothelial cell
ECM	Extracellular matrix
ESC	embryonic stem cell
FGF	Fibroblast growth factor
IGF	Insulin growth factor
GelMA	Gelatin-Methacryloyl
GO	Graphen oxide
H9c2	Cardiomyoblasts
HF	Heart failure
IHD	Ischaemic heart disease
iPSCs	Induced pluripotent stem cells
MeTro	Methacrylated tropoelastin
MHC	Myosin heavy chain
MLC	Myosin light chain
MSC	Mesenchymal stem cell
NF	Nanofibrous
NP	Nanoparticle
NR	Nanorod
NW	Nanowire
PANI	Polyaniline
PCL	Poly(ε-caprolactone)
PEG	Polyethylene glycol
PEGDA	Poly(ethylene glycol) diacrylate
PEOz	Poly (2-ethyl-2-oxazoline)
PGA	Poly glycolic acid
PGS	Poly(Glycerol Sebacate)
PHB	Poly(3-hydroxybutyrate)
PLCL	Poly (L-lactide-co-caprolactone)
PLGA	Poly(lactic-co-glycolic acid)
PLL	Poly L-lysine
PLLA	Poly(L-lactic acid)
PMGI	Polydimethylglutarimide
PPS	Poly(propylene sulfide)
PS	Polystyrene
PPy	Polypyrrole
PTh	Polythiophene
PVA	Poly(vinyl alcohol)
SC	Stem cell
SF	Silk Fibroin
TGF-β1	Transforming growth factor-β1
TiO_2_	Titanium dioxide
TMC	N,N,N-trimethylchitosan chloride
VEGF	Vascular endothelial growth factor
α-SA	α-sarcomeric actinin
